# Evaluating the Effectiveness of Educational Interventions in Family Planning for Men in Developing Countries: A Systematic Review

**DOI:** 10.31662/jmaj.2023-0018

**Published:** 2023-11-16

**Authors:** Haruko Tazoe, Riho Tomozawa, Mai Sato, Sumire Anzai, Rikuya Hosokawa

**Affiliations:** 1 Department of Human Health Sciences, Graduate School of Medicine, Kyoto University, Kyoto, Japan

**Keywords:** contraceptives, developing countries, family planning

## Abstract

**Introduction:**

Unintended pregnancy is associated with national socioeconomic development and gender inequality. In addition to contraception, educational interventions that promote family planning and address gender dynamics are considered important in preventing unintended pregnancy. While the importance of encouraging men’s participation in family planning has been advocated, most studies have focused on the application of interventions to women or populations in high-income countries only. Therefore, we conducted a systematic review to evaluate the effects of educational interventions on men in low- and middle-income countries in terms of knowledge, attitudes, practices, and gender dynamics.

**Methods:**

Three electronic databases (CINAHL, Ovid MEDLINE, and Web of Science) were searched for studies published from January 1980 to October 2022. Keywords such as “men/husband,” “family planning,” “contraception,” and “education” were combined to identify studies. Two independent reviewers conducted screening and data extraction, and the risk of bias was assessed using the Risk of Bias 2 tool. The quality of evidence was evaluated according to the GRADE Handbook.

**Results:**

The database search identified 16,086 articles, of which 4 cluster randomized controlled trials (RCTs) and 1 RCT were ultimately included. Each of them was conducted in four different countries: Malawi, Guatemala, Tanzania, and India. Changes in knowledge, attitude, family planning, and gender dynamics were the outcomes used to assess the effectiveness of interventions. The five selected articles exhibited an effect on ≥1 indicator for each outcome. However, the quality of evidence was determined to be low or very low owing to the risk of bias, heterogeneity, and imprecision.

**Conclusions:**

Determining the effectiveness of educational interventions in family planning for men in low- and middle-income countries requires additional high-quality intervention studies. As family planning is influenced by various background factors, it is important to develop appropriate interventions for each context and define relevant indicators that can be compared across contexts.

## Introduction

Unintended pregnancy is associated with social and economic development and gender equality in a country ^(1)^; hence, the inclusion of men in family planning is imperative ^(2)^. Indeed, unintended pregnancies occur worldwide, with an estimated 121 million cases annually ^(3)^.

Unintended pregnancy is defined as “a pregnancy occurring to a woman who was not planning to have any (more) children, or that was mistimed, in that it occurred earlier than desired ^(1)^.” According to Bearak et al., the average rate of unintended pregnancy worldwide was estimated to be 64 per 1,000 live births among women of reproductive age (15-49 years) in the years 2015-2019 ^(3)^. Although this average has been improved from the estimated unintended pregnancy rate of 79 reported during 1990-1994, disparities exist when regional and socioeconomic conditions are considered. The World Bank estimates rates of 34, 66, and 93 unintended pregnancies per 1,000 live births in high-, middle-, and low-income countries, respectively, indicating an inverse relationship between unintended pregnancy and national income ^(2)^.

Supporting sexual and reproductive health and achieving gender equality are part of the United Nations’ Sustainable Development Goals and the United Nations Population Fund, a subsidiary agency of the United Nations that works to ensure reproductive rights for all and supports access to a wide range of sexual and reproductive health services ^(1)^. Specific measures to combat unintended pregnancy have been attempted through material means, including the provision of contraceptives and contraceptive pills and expansion of contraceptive services ^(1)^. While contraceptive use is increasing worldwide ^(4), (5)^, progress may vary between countries. According to Kantorová et al., contraceptive needs are not being met in developing regions, which have an estimated 214 million people of reproductive age ^(4)^. Therefore, providing access to contraceptives in populations with unmet needs can prevent unintended pregnancies. However, rather than the lack of access to contraception, barriers, including the lack of knowledge, concerns regarding side effects, social stigma, and opposition from partners and others, are the major obstacles to contraceptive use in low- and middle-income countries ^(1), (5), (6), (7)^. Therefore, contraceptive coverage alone is insufficient to reduce the rates of unintended pregnancy; educational interventions, such as promoting family planning and addressing underlying social norms, including gender dynamics, are also essential ^(5)^.

Family planning can be defined as “the information, means, and methods that allow individuals to decide if and when to have children ^(1)^.” It can prevent unintended pregnancies or pregnancies in young girls, increase educational opportunities, empower women and girls, and promote a sustainable population growth ^(1), (8)^. The incidence of unintended pregnancy is closely related to gender inequality. Thus, gender dynamics are important in family planning ^(1), (9)^; it is crucial to promote men’s participation in family planning and establish communication between couples and partners to promote family planning ^(2)^.

Previous studies have reported that approximately 59% and 42% of men in rural Ethiopia and in pastoralist communities, respectively, are involved in family planning ^(10), (11)^. Furthermore, 48% of men in rural Ghana reportedly participate in family planning services ^(12)^. Factors related to men’s participation in family planning include communication with their spouses concerning sexual and reproductive health, family planning, and agreement with family planning ^(10), (11)^. In addition, a systematic review in the Philippines found that the most common barrier to male participation in family planning was the lack of knowledge regarding the proper use of contraception and its side effects ^(13)^.

Educational interventions can promote family planning. A cluster randomized controlled trial (RCT) based on pastoralist communities in Ethiopia reported that education among men regarding family planning had promoted family planning use and attitudes toward family planning ^(14)^. An RCT involving married women in Jordan reported an increase in modern contraception use in the group that underwent educational counseling for couples compared with the group that did not ^(15)^. However, the impact of education on men remains unclear as these studies have mainly focused on women; their responses were applied to assess impact. An RCT of a fishing community in Uganda reported that education regarding family planning using easy-to-understand handouts increased knowledge and the use of family planning methods ^(16)^. However, because that study included both men and women, the results were mixed, and the impact of education on men was unclear. A systematic review of the effects of educational interventions focused on contraception in family planning showed that while various educational approaches can increase contraceptive knowledge, there is insufficient research to evaluate their effects on contraceptive attitudes, selection of effective methods, and contraceptive use. Although the included studies had high quality ^(17)^, they were conducted in high-income countries, with only a few focused on men in low- and middle-income ones.

Therefore, we conducted a comprehensive systematic review of the effectiveness of educational interventions on family planning in low- and middle-income countries, particularly focusing on their impact on men.

## Materials and Methods

The study was conducted based on the Preferred Reporting Items for Systematic Reviews and Meta-Analyses (PRISMA). Particularly, the Japanese version of the checklist translated by Hiroharu et al. was used in this study ^(18)^. The English version is listed in Appendix 1. The systematic review was registered with PROSPERO (CRD42022374221), and no protocol article was published.

### Inclusion/Exclusion criteria

We included primary quantitative RCTs or cluster RCTs published in English after 1980. Promotion of family planning programs began at the International Conference on Population and Development in 1974, and the concept of sexual and reproductive health/rights was introduced in 1994. These worldwide discussions on family planning as well as sexual and reproductive health/rights have led to increased research on these topics since the 1980s. Studies not falling under the above categories and those in the form of review articles, conference proceedings, and protocols were excluded.

We included men from low- and middle-income countries (i.e., countries with Gross National Income < $13,205 based on the World Bank’s Gross National Income per capita income classification) ^(19)^. Men from high-income countries were excluded. No restrictions were imposed on the number of children, history of contraceptive use, history of unintended pregnancy by the partner, or education on family planning before the intervention. We excluded interventions aimed at preventing sexually transmitted diseases (STDs) or the recurrence of STDs in populations at a high risk of STDs. Sex workers, sexual minorities, and men from low- and middle-income countries who had migrated to high-income ones were excluded.

We included all educational interventions based on family planning, contraception, and sexual and reproductive health (i.e., interventions aimed at promoting family planning practices, such as contraceptive use, and improving attitudes toward family planning). No restrictions were imposed on the intervention format (remote/face-to-face), duration, or providers. Interventions targeting couples or communities that included men were also included, as were interventions targeting men alone.

As regards the comparison of this review, we did not impose a limitation on a specific population as a comparison group. However, studies without a comparison group were excluded.

As regards the outcomes of this review, we focused on knowledge, attitudes, and practices concerning family planning and gender dynamics in decision-making. These outcomes were set with reference to two systematic reviews that evaluated the effects of educational interventions on family planning ^(17), (20)^ and explored the factors associated with family planning in low- and middle-income countries ^(6)^. Gender dynamics refer to the relationships and interrelationships between men and women.

### Search strategy

Electronic database searches were conducted on the CINAHL, Ovid MEDLINE, and Web of Science (last search date: 2022/10/11). The search term was a combination of keywords related to men and spouses (men, partner, husband) as terms for the target population; keywords related to sex education and family planning (such as sex education, family planning, contraception, and birth control) as terms for the intervention type; and keywords related to education (education, intervention). Furthermore, we used a combination of keywords related to sex education (sex education), family planning (family planning, contraception, birth control), and education (education, intervention) as terms to indicate the study design. Furthermore, keywords related to the study design (RCT, clinical trials) were used. We referred to the search formulas of Pazol et al., Sharma et al., and King’s College London’s “Searching for Systematic Reviews: Advanced Search Techniques” to develop the search formulas ^(17), (20), (21)^. The search formulas are presented in Appendix 2.

### Study selection

Five reviewers performed the study selection. First, electronic databases were searched using a search formula, and the literature from each database was imported into Covidence (https://app.covidence.org; Covidence Inc., Melbourne, Australia), a tool for systematic reviews. Duplicate articles were excluded. Titles and abstracts were screened, followed by full-text screening by two independent reviewers. The full-text screening was performed in two stages, with the first stage focusing on the population and intervention-based content and the second focusing on outcomes. Disagreements were resolved through discussion between the two reviewers, and when disagreements persisted, a third reviewer was included to resolve them.

### Data extraction

Data were extracted independently by two reviewers. A format created in Microsoft Excel (Microsoft Corp., Redmond, WA) was used for data extraction and organization. The following data were extracted: study author, year, study country, study design, study objectives, population characteristics (eligibility criteria, characteristics, sample size), randomization process, intervention group/comparison group characteristics (implementer, duration, composition), outcomes (indicators, measurement methods), data analysis, main results, limitations, and implications for the future.

### Risk-of-bias assessment

Risk-of-bias assessment was independently conducted by two reviewers. The Cochrane Risk of Bias 2 tool, which has models for RCTs and cluster RCTs, is available on the Web as an open resource ^(22)^. The risk-of-bias assessment items for bias included five domains for RCTs: bias arising from the randomization process, bias owing to deviations from the intended intervention, bias owing to missing outcome data, bias in the measurement of outcomes, and bias in the selection of reported outcomes. For cluster RCTs, aside from the five domains of RCTs, the domain of bias owing to the timing of participant identification or recruitment in cluster randomization was also included. Based on these assessments, the reviewers judged the bias on a 3-point scale of “low,” “some concerns,” and “high,” with any disagreements resolved by discussion between the two reviewers. A narrative synthesis was used to integrate the results.

### Quality-of-evidence assessment

The GRADE Handbook ^(23)^ was consulted to assess the overall quality of evidence of all the included studies. As we focused on RCTs and cluster RCTs as the study design of interest, the level was reduced by one or two when there was a risk of bias, inconsistency, indirectness of evidence, imprecision, or publication bias and finally rated as “high,” “moderate,” “low,” or “very low.”

## Results

**Figure 1.**
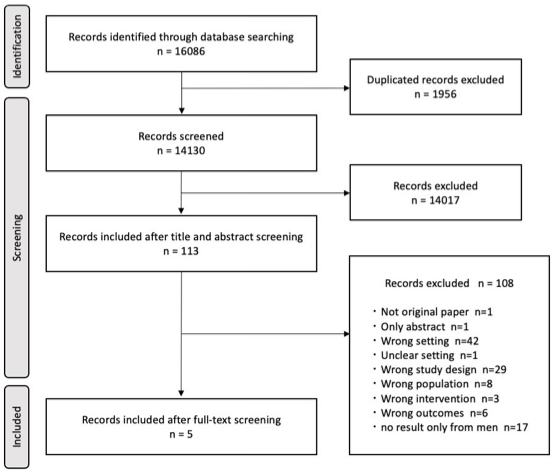
PRISMA flowchart.

Overall, 16,086 articles were identified during the database search. Of these, 1,956 were excluded because of duplicates, resulting in 14,130 articles eligible for screening. The title and abstract screenings excluded 14,017 articles as they did not meet the eligibility criteria, and 113 articles were included in the full-text screening. The full-text screening excluded 108 articles; thus, 5 articles were finally included in the study. The reasons for exclusion, ranked by priority at the time of screening, were as follows: not an original article, abstract only, different setting (high-income country), setting unknown, different study design, different population, different intervention, different outcome, and results obtained only from men. Figure 1 presents the process of study selection.

Table 1 provides an overview of the five included studies ^(24), (25), (26), (27), (28)^. The articles were published after 2011, and the study designs included one RCT ^(24)^ and four cluster RCTs ^(25), (26), (27), (28)^. The included studies were conducted in four countries: Malawi ^(24)^, Guatemala ^(25), (28)^, Tanzania ^(26)^, and India ^(27)^. Table 2 provides additional information of each country. Three of the included studies were conducted in low-middle-income countries ^(26), (27), (28)^, one in a low-income country ^(24)^, and one in an upper-middle-income country ^(25)^, according to the World Bank Income Classification 2022 ^(19)^. The total fertility rate was the sum of age-specific fertility rates for women aged 15-49 years, and the fertility rate was the ratio of the number of births per 1,000 individuals ^(29), (30), (31)^. When considering the gender gap, which is an index of gender parity across key dimensions (Economic Participation and Opportunity, Educational Attainment, Health and Survival, and Political Empowerment), Tanzania had the lowest gender gap, followed by Guatemala, Malawi, and India ^(32)^.

**Table 1.** Summary of Studies Included.

Author, Year	Design	Setting	Objective	Population: Inclusion/Exclusion	Intervention/Comparison	Outcome
Shattuck, 2011 ^(24)^	RCT	Malawi; Mangochi	To evaluate the effectiveness of peer education intervention based on the information-motivation-behavioral skills (IMB) model on contraceptive use among couples.	397 men Inclusion: Individuals aged 18-25 years, married or cohabiting with a woman who is not pregnant/breastfeeding a child aged <6 months, no infertility treatment in the past 3 months, no use of modern contraception Intervention population (baseline→endpoint): 197→149 (men) Comparison population (baseline→endpoint): 200→140 (men) Religion: Christian and Muslim	Intervention: Peer educational intervention on family planning (Malawi Male Motivator Project) [Contents] Home visits (five times) by trained male motivators: providing information on family planning, sharing experiences, motivating, role-playing, and improving communication skills/6 months Comparison: None	Index: Contraceptive use, knowledge, attitudes, self-esteem, gender norms, communication regarding family planning, communication frequency Assessment: Questionnaire Timepoints: ①baseline ②within 1 month of intervention
Schuler, 2015 ^(25)^	cluster-RCT	Guatemala; Sacatepéquez, Chimaltenango, Sololá, Huehuetenango, San Marcos; (rural area)	To investigate the effectiveness of interventions in rural areas where contraceptive coverage is low and family planning needs are unmet compared with urban areas.	45 communities (15 communities × 3→ a community has gone) Inclusion: Married men or in civil union, wives aged 18-40 years, live in the districts, agreement from both members of couples Intervention population: 597→328 (men and women) Comparison population: 488→213 (men and women) Religion: Catholic 50%-57%, Protestant 31%-38%.	Intervention: Workshop Session (expressed as “WS” in the following) [Contents] WS by trained local promoters: gender issues presenting barriers to sexual and reproductive health, information and role-play on gender equality and family planning/WS for men × 2, WS for women × 2, WS for couple × 2 Comparison: None	Index: Gender and Family Planning Equity [GAFPE] Scale: Attitudes toward gender equality, family planning knowledge Assessment: Interview Timepoints: ①baseline ②2 months after baseline
Mmbaga, 2017 ^(26)^	cluster-RCT	Tanzania; Kinondoni Municipality	To evaluate the effectiveness of interventions to delay the initiation of sexual activity and promote the use of condoms.	38 schools: 5,091 students Inclusion: Students aged 12-14 years, able to read and write Intervention population: 2,503→2,134 (boys and girls) Comparison population: 2,588→2,236 (boys and girls) Religion: Christian and Muslim, respectively, approximately 50%	Intervention: Three-component sessions with peers, teachers, and healthcare professionals (PREPARE) [Contents] Teachers: In-class interactive teaching and learning sessions; Peers: one session per week for a total of nine life skills training sessions; health professionals: sessions on accessing sexual and reproductive health services and contraceptive use Comparison: None	Index: An action plan for sexual activity, sexual initiation, and condom use Assessment: Questionnaire Timepoints: ①baseline ②6 months after intervention (→booster intervention) ③12 months after intervention
Fleming, 2018 ^(27)^	cluster-RCT	India; Thane (rural area)	To evaluate the effects of the CHARM intervention on gender ideology and household decision-making.	50 clusters: 1,081 couples Inclusion: Married men aged 18-30 years, husbands and wives fluent in Marathi, resided together for the past 3 months with no intent to relocate in the next 2 years. Exclusion: couples who report infertility or surgical sterilization exhibiting severe cognitive or health impairment. Intervention population: 469→397 (couples) Comparison population: 612→494 (couples) Religion: Designated tribes were the most common (66.1%).	Intervention: Counseling on family planning and gender equality (CHARM) [Contents] Counseling sessions with trained village male healthcare providers/sessions (expressed as “S” in the following) for men × 2, S for couple × 1: Providing information on family planning, decision-making related to family planning, gender equality, etc Comparison: Public family planning services	Index: Gender Equitable Men (GEM) Scale: gender ideology, equitable attitudes toward household decision-making Assessment: Questionnaire Timepoints: ①baseline ②9 months after baseline ③18 months after baseline
Waidler, 2022 ^(28)^	cluster-RCT	Tanzania; Mufindi, Mafinga, Busokelo, Rungwe	To examine the impacts of a multicomponent intervention targeted to Tanzanian adolescents on their sexual behaviors and reproductive health.	130 clusters: 1,993 individuals (1,065 men) Inclusion: All adolescents aged 14-17 in PNNS households (Productive Social Safety Net program [PNNS]; “Cash”), both in and out of school, were included. Intervention population: 1,272→1,128 (boys and girls) Comparison population: 1,186→1,063 (boys and girls).	Intervention: Cash, livelihood, and life skills training & training on HIV and SRH [Contents] Livelihoods and life skills training, mentoring and asset transfer, strengthening youth-friendly HIV and SRH services: topics on contraception, access to gender services, and gender/sessions with online educational curriculum and breakout rooms Comparison: Cash only	Index: Contraceptive knowledge, modern contraceptive knowledge, condom use, modern contraceptive use Assessment: Questionnaire Timepoints: ①baseline ②2-3 months after intervention ③17 months after intervention

**Table 2.** Supplementary Information of Each Country.

Country	Income class	Population	Fertility rate	Birth rate	Gender gap
Guatemala (Schuler, 2015)	upper-middle	16,858	2.5	22	0.664 (113th)
India (Fleming, 2018)	lower-middle	1,396,387	2.1	17	0.629 (135th)
Tanzania (Mmbaga, 2017) (Waidler, 2022)	lower-middle	61,704	4.8	37	0.719 (64th)
Malawi (Shattuck, 2011)	low	19,377	4.0	33	0.632 (132nd)

As regards the randomization unit, three community-based cluster RCTs ^(25), (27), (28)^, one school-based cluster-RCT ^(26)^, and one individual-based RCT ^(24)^ were included. Three, one, and one studies included married or partnered individuals aged ≥18 years ^(24), (25), (27)^, school-going or out-of-school adolescents aged 14-19 years ^(28)^, and school-going students aged 12-14 years, respectively ^(26)^.

Two interventions ^(24), (25)^ were delivered by peers living in the study area. In other studies, interventions were delivered by local healthcare providers ^(27)^, a combination of people (including peers, teachers, and healthcare providers) ^(26)^, or by an online curriculum ^(28)^. Most interventions were performed by trained local personnel as the providers. Most interventions included informative and interactive educational sessions and role-playing activities regarding family planning, gender equality, and sexual and reproductive health, whereas other interventions included improvement in communication skills and livelihood/life skills training combined with cash transfer incentives.

Comparisons were made with groups receiving no intervention (three studies) ^(24), (25), (26)^, only referrals to public family planning services (one study) ^(27)^, and only cash benefits (one study) ^(28)^. The outcomes were categorized into knowledge, attitude, practice, and gender dynamics of family planning. Four outcome measures were extracted for knowledge: knowledge of contraception, knowledge limited to modern contraception, and knowledge of family planning. Outcome measures for attitude included attitude toward family planning, self-efficacy, communication with partners about family planning, and action plans for delaying sexual activity and condom use. Practice outcomes included indicators of contraceptive use, condom use, and initiation of sexual activity. Gender dynamics outcomes included gender norms and fairness in decision-making within the household. Outcomes were measured once or twice following the intervention, and the timing varied from 2 months after the baseline survey to 18 months. The results of the risk-of-bias assessment for each outcome are presented in Table 3.

**Table 3.1.**
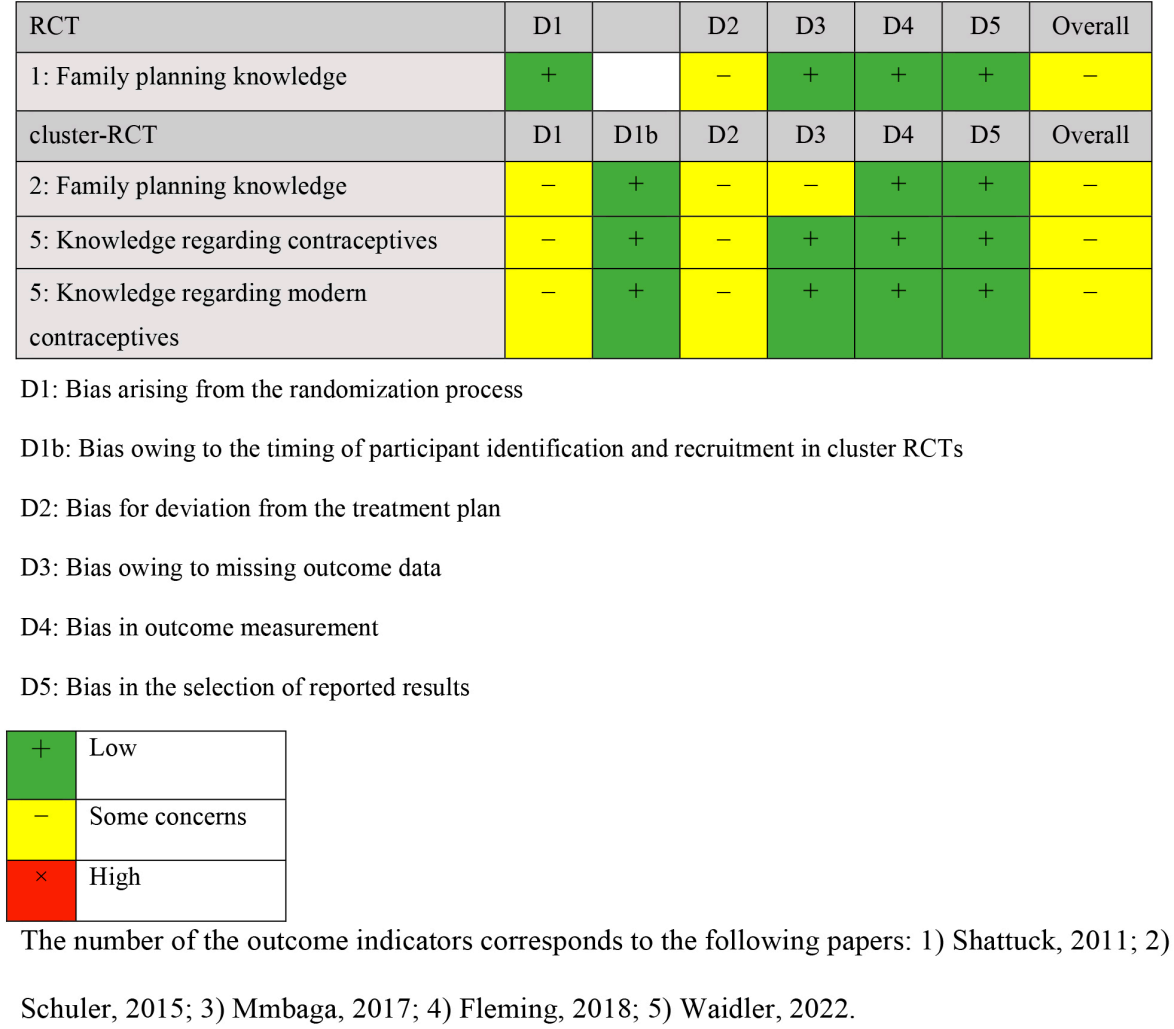
Risk-of-Bias Assessment: Knowledge.

**Table 3.2.**
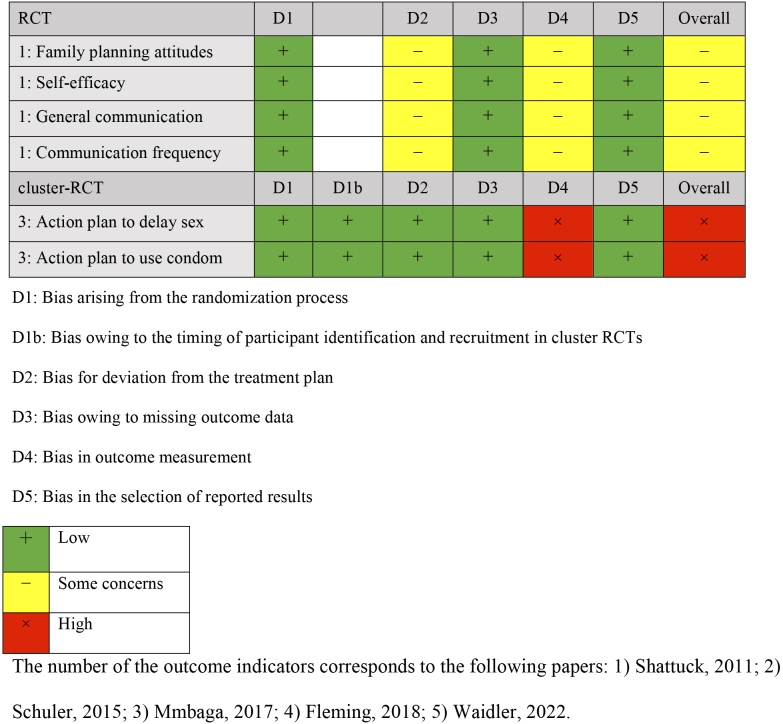
Risk-of-Bias Assessment: Attitude.

**Table 3.3.**
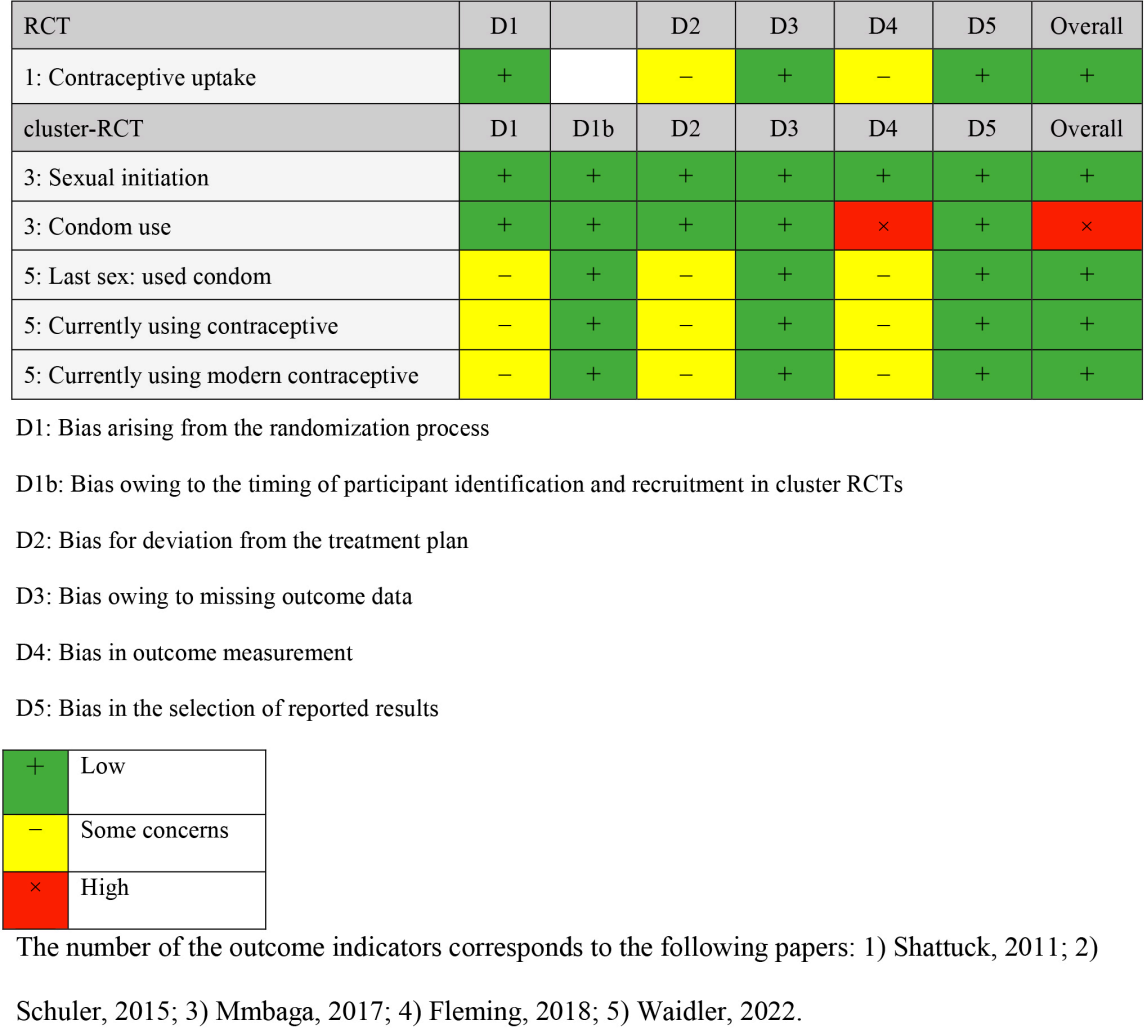
Risk-of-Bias Assessment: Practices.

**Table 3-4.**
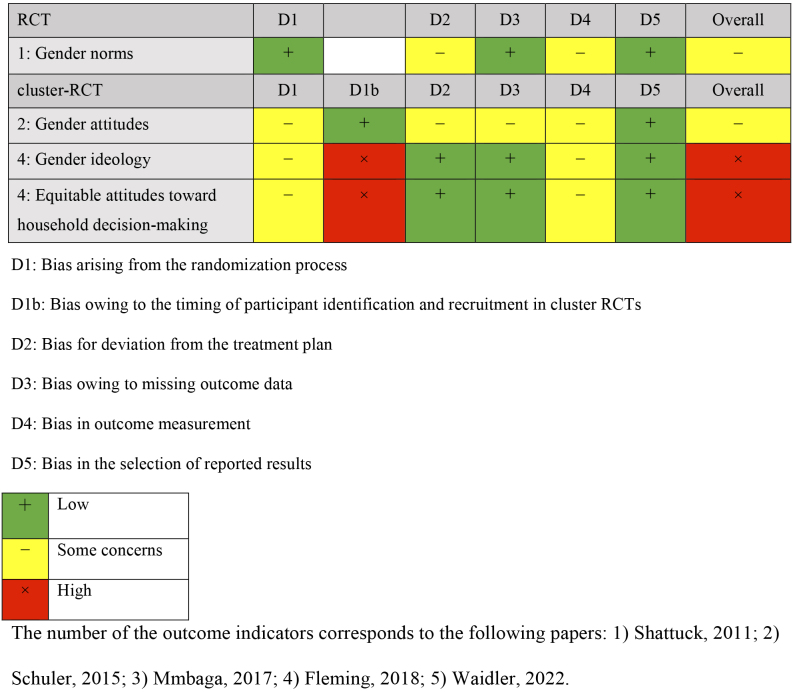
Risk-of-Bias Assessment: Gender Dynamics.

Table 4 summarizes the results of the effectiveness of the interventions. Outcome measures were described as they appeared in each study as follows: 1) Knowledge: One of the three studies examining the effect of educational interventions on knowledge demonstrated statistically significant improvement in one outcome measure (family planning knowledge). The study targeted the community and included interventions for men and women, as well as for couples ^(25)^. The intervention comprised informational and role-playing sessions. After 2 months, the number of known contraceptive methods increased. The risk of bias in the measure was assessed as “some concern.” 2) Attitude: One of the two studies examining the effects of educational interventions on attitude demonstrated a statistically significant improvement in one outcome measure (communication frequency). The study targeted men, and peer support on contraception was provided as motivation and information ^(24)^. The questionnaire at 6 months reported a significant increase in the frequency of communication regarding family planning with their partner. The risk of bias in the measure was assessed as “some concern.” 3) Practice: Two of the three studies examining the effects of educational interventions on practice demonstrated statistically significant improvements in a total of three outcomes measured: sexual initiation, condom use, and contraceptive uptake. The school-based intervention reported significant differences in condom use and age of sexual initiation at 12 months ^(26)^. Interventions targeting men of reproductive age also reported significant increases in contraceptive uptake ^(24)^. The risk of bias was rated as “low” for sexual initiation and contraceptive uptake and “high” for condom use. 4) Gender dynamics: Two of the three studies that examined the effects of educational interventions on gender dynamics found statistically significant improvements in two outcome measures: equitable attitudes toward household decision-making and gender attitudes. An intervention on family planning and gender ideology for couples reported a significant improvement in “Equitable attitudes toward household decision-making” at 9 months ^(27)^. Conversely, no significant increase was observed at 18 months, indicating that the effect was not sustained. Interventions for communities reported significant increases in scores for equal attitudes toward gender ^(25)^. The risk-of-bias assessment in the outcome measures was “high” for equitable attitudes toward household decision-making and “some concerns” for gender attitudes. Because the study design for all outcomes was RCT or cluster-RCT, the GRADE rating was reported as “high.”

**Table 4.** Summary of Findings.

Outcome	Publication	Impact of intervention	*p*-value
Knowledge	Waidler, 2022	Knowledge regarding contraceptives	*p* ≥ 0.05
		Knowledge regarding modern contraceptives	*p* ≥ 0.05
	Schuler, 2015	Family planning knowledge*	*p* < 0.001
	Shattuck, 2011	Family planning knowledge	*p* < 0.05
Attitude	Mmbaga, 2017	Action plan to delay sex	*p* = 0.614
		Action plan to use a condom	*p* = 0.0876
	Shattuck, 2011	Family planning attitude	*p* ≥ 0.05
		Self-efficacy	*p* ≥ 0.05
		General communication	*p* ≥ 0.05
		Communication frequency*	*p* < 0.05
Practice	Waidler, 2022	Used a condom during last sexual encounter	*p* ≥ 0.05
		Currently using contraceptives (among sexually active)	*p* ≥ 0.05
		Currently using modern contraceptives (among sexually active)	*p* ≥ 0.05
	Mmbaga, 2017	Sexual initiation*	*p* = 0.043
		Condom use*	*p* = 0.004
	Shattuck, 2011	Contraceptive uptake*	*p* < 0.05
Gender Dynamics	Fleming, 2018	Sex ideology	*p* ≥ 0.05
		Equitable attitudes toward household decision-making*	*p* < 0.01 (9 months) *p* ≥ 0.05 (18 months)
	Schuler, 2015	Sexual attitudes*	*p* = 0.002
	Shattuck, 2011	Sexual norms	*p* ≥ 0.05

Note: *Significant differences between the intervention and comparison groups (*p* < 0.05)

**1) Knowledge:** According to the GRADE Handbook, the three sources of evidence reporting knowledge were rated as “very low” with downgrades for risk of bias, imprecision, and inconsistency. First, the risk of bias was downgraded by one grade to “moderate” as all outcome measures were rated as “some concerns.” Inaccuracy was downgraded by one grade to “low” as it included articles for which 95% confidence intervals (CIs) could not be accurately evaluated. Heterogeneity was downgraded to “very low” as it was difficult to interpret the consistency of the effects. Indirectness was not determined because no population or outcome showed indirectness. Publication bias was not determined as the number of studies was insufficient for evaluation. **2) Attitude:** The two pieces of evidence reported on attitudes were evaluated holistically and downgraded to “very low” for risk of bias, imprecision, and inconsistency. First, the risk of bias was downgraded by two grades to “low” as two outcome measures were rated as “high” and four as “some concerns.” The risk of inaccuracy and inconsistency was downgraded to “very low” for the same reasons as above. Publication bias was not determined owing to the insufficient number of studies. **3) Practice:** The three studies were rated as “low” with downgrades for imprecision and inconsistency. First, concerning the risk of bias, one outcome measure was rated as “high,” whereas the other five were rated as “low” and, thus, were downgraded based on the GRADE Handbook guidlines that most information across studies comes from studies with low risk of bias. Therefore, downgrading was not performed. Next, the imprecision was downgraded by one grade to “moderate” because the study included articles for which 95% CIs could not be accurately evaluated. Heterogeneity was downgraded to “low” as it was challenging to interpret the consistency of the effects. Indirectness was downgraded to “low” because no population or outcome showed nonindirectness, and publication bias was not determined because of the insufficient number of studies. **4) Gender dynamics:** The three evidence sources reported on gender dynamics were holistically evaluated and downgraded to “very low” for risk of bias, imprecision, and inconsistency. The risk of bias was downgraded by two grades to “low” given that two outcome measures were rated as “high” and two as “some concerns.” The risk of inaccuracy and inconsistency was downgraded to “very low” for the same reasons as above. Publication bias was not determined because of the insufficient number of studies.

## Discussion

This systematic review investigated the effects of educational interventions on family planning for men in low- and middle-income countries and evaluated outcomes, including changes in family planning knowledge, attitudes, practices, and gender dynamics. Five articles were selected for the review, including four based on couples and one based exclusively on men that exhibited effects on ≥1 indicator for each outcome. However, the evidence quality was rated as “very low” for knowledge, attitudes, and gender dynamics and “low” for practice because the heterogeneity of the measures did not allow for a consistent evaluation of the intervention effects, bias risk remained, and data, including CIs, were insufficiently described.

Community-based approaches to educational interventions have yielded positive results, as demonstrated by Schuler et al ^(25)^. According to findings from this review, community education on family planning can contribute to increased family planning knowledge ^(20)^. However, the study by Schuler et al. may have been limited to rural areas, where contraceptive use is low and family planning needs are unmet compared with urban areas. Indeed, approaching groups with unmet contraceptive needs enhances the promotion of contraceptive use ^(5)^; hence, we believe that approaching groups with high needs is more effective in terms of intervention. A study of fishing communities in Uganda, where family planning needs are considered high, found an increase in knowledge among men and women, which suggests that community education for high-need groups has the potential to improve knowledge; however, in-depth studies are warranted.

Shattuck et al. reported that interventional effectiveness was obtained for attitude; Mmbaga et al. and Shattuck et al. reported that practice was the outcome of their respective interventions. By outcome, more effect indicators were obtained for practice than for attitude ^(24), (26)^. The behavioral change stage model suggests that attitudes change from a period of indifference to a period of interest, followed by behavioral change ^(33)^. Therefore, it can be inferred that attitude change is observed before behavioral change. However, our findings did not conform to this assumption. The first reason is that these outcome measures were not obtained from the same study. Second, the age and marital status of the participants and timing of outcome measure differed in each study. Thus, the results may differ depending on the characteristics of the target population. Third, all studies used self-reported measures of practice, which may have been more effective than other outcome measures as they used simple measures such as using family planning methods and were also considered to be more directly affected by intervention content. In low- and middle-income countries, increased knowledge of family planning positively impacts other aspects of contraceptive use, including positive attitudes toward contraception and contraceptive use retention ^(17)^. While the effects of knowledge, attitudes, and implementation are important, exploring their interactions will create more effective educational interventions to promote behavioral change in family planning in the future.

Fleming et al. and Schuler et al. reported the effects of educational interventions on gender dynamics and included couples ^(25), (27)^. According to Tilahun et al., educational interventions on family planning, such as including couples and promoting communication between spouses, can help promote contraceptive practices ^(34)^. Furthermore, a systematic review of behavioral change techniques suggests that the most effective interventions involve the male partner in contraceptive decision-making and combine multiple behavioral change techniques ^(35)^. Therefore, interventions involving men and couples can affect gender dynamics, which is a fundamental aspect of the decision-making process. However, according to Ruane-McAteer et al., evidence on intervention studies to improve gender inequalities in sexual and reproductive health and rights is generally of low quality and inconclusive ^(36)^. Thus, further evidence on the effects of interventions on gender dynamics in family planning is needed.

### Strengths and limitations

To the best of our knowledge, this is the first review to examine the effects of educational interventions on family planning in low- and middle-income countries by focusing on men. In addition, few systematic reviews have explored the impact of interventions in terms of gender dynamics in the decision-making process as an outcome. Furthermore, the study design was limited to RCTs or cluster RCTs, which allowed us to investigate the effects of interventions based on data obtained from the randomization process, thereby reducing bias, and is a rigorous tool examining the cause-and-effect relationships.

However, this study has certain limitations. First, the search was limited to three electronic databases and articles published in English; thus, some articles might have been missed. Second, the number of studies eligible for inclusion was small, with only two or three studies for each outcome. Thus, the obtained data could be insufficient to conduct subgroup analysis/meta-analysis, and the study findings cannot be generalized to whole target populations. Finally, knowledge, attitudes, and practices related to family planning and gender dynamics, which were set as outcomes in this study, can be affected by religious, cultural, and economic factors besides education ^(1), (37)^. Therefore, the findings should be carefully interpreted based on the characteristics of the setting and target population.

### Conclusion

It is necessary to conduct more high-quality intervention studies that consider various factors and generate evidences through more comprehensive searches in the future. As various background factors influence family planning, it is important to develop appropriate interventions in each context. In this work, the findings suggest that interventions targeting high-need populations and educational interventions that include community and couple interventions may be helpful.

In addition to developing interventions that fit various contexts, it is important to provide comprehensive evidence in low- and middle-income countries. Thus, it is necessary to develop consistent and appropriate outcome measures that can be adapted across diverse contexts to ensure accuracy in reporting.

## Article Information

### Conflicts of Interest

None

### Sources of Funding

The research was conducted using research funds from the Kyoto University.

### Acknowledgement

We are grateful to the Kyoto University for providing research funds to conduct this study.

### Author Contributions

 HT acquired the funds needed for the study; was involved in finalizing the methodology, administering the project, acquiring the necessary resources, and securing the software required for the data analysis; and also drafted the original manuscript. RT, MS, SA, and RH reviewed and edited the manuscript and provided supervision. HT, RT, MS, SA, and RH conducted the investigations and performed study validation and visualization. All the authors read and approved the final manuscript.

### Approval by Institutional Review Board (IRB)

This review has not undergone ethical review.

## Supplement

Appendix 1

Appendix 2
