# Supplementary material for: Evaluating the Effectiveness of Educational Interventions in Family Planning for Men in Developing Countries: A Systematic Review
Source: JMA J. 2023 Nov 16;7(1):40–51. doi: 10.31662/jmaj.2023-0018 (PMC10834174; doi:10.31662/jmaj.2023-0018)
Supplement: Appendix 2 [file 2433-3298-7-1-0040-s002.pdf]

Appendix 2. Database search strategy

Ovid MEDLINE	Web of Science	CINAHL
1. Male/<br>2. Men/<br>3. Sexual Partners/<br>4. Spouses/<br>5. Fathers/<br>6. Partner*<br>7. Spouse*<br>8. Father*<br>9. Husband*<br>10. Dad	1. Male<br>2. Men<br>3. "Sexual Partners"<br>4. Spouses<br>5. Fathers<br>6. Partners<br>7. Husbands<br>8. Dad	1. "Male"<br>2. "Men+"<br>3. "Sexual Partners"<br>4. "Spouses"<br>5. "Fathers+"<br>6. "Partner*"<br>7. "Spouse*"<br>8. "Father*"<br>9. "Husband*"<br>10. "Dad"
11. 1 OR 2 OR 3 OR 4 OR 5 OR 6 OR 7 OR 8 OR 9 OR 10	9. 1 OR 2 OR 3 OR 4 OR 5 OR 6 OR 7 OR 8	11. 1 OR 2 OR 3 OR 4 OR 5 OR 6 OR 7 OR 8 OR 9 OR 10
12. Sex Education/<br>13. Sexual adj2 education<br>14. Sexual adj2 intervention<br>15. Sexuality education	10. "Sexual NEAR/2 education"<br>11. "Sexual NEAR/2 intervention"<br>12. "Sexuality education"<br>13. "Sex education"	12. "Sex Education"<br>13. Sexual N2 education<br>14. Sexual N2 intervention<br>15. "Sexuality education"
16. 12 OR 13 OR 14 OR 15	14. 10 OR 11 OR 12 OR 13	16. 12 OR 13 OR 14 OR 15
17. Contraception/<br>18. Contracept*<br>19. Family planning services/<br>20. Family planning<br>21. Pregnancy/<br>22. Pregnan*<br>23. Birth control<br>24. Parenthood<br>25. Reproductive health/<br>26. Reproductive health<br>27. Sexual health/	15. Contraception<br>16. Contracept?<br>17. Pregnancy<br>18. Pregnan?<br>19. "Birth control"<br>20. Parenthood<br>21. "Reproductive health"<br>22. "Family planning"<br>23. "Sexual health"	17. "Contraception+"<br>18. Contracept*<br>19. "Pregnancy+"<br>20. Pregnan*<br>21. "Birth control"<br>22. "Parenthood+"<br>23. "Reproductive health"<br>24. "Reproductive health"<br>25. "Family planning+"<br>26. "Sexual health"
28. 17 OR 18 OR 19 OR 20 OR 21 OR 22 OR 23 OR 24 OR 25 OR 26 OR 27	24. 15 OR 16 OR 17 OR 18 OR 19 OR 20 OR 21 OR 22 OR 23	27. 17 OR 18 OR 19 OR 20 OR 21 OR 22 OR 23 OR 24 OR 25 OR 26
29. Health education/<br>30. Education/<br>31. Intervention	25. "Health education"<br>26. Education<br>27. Intervention	28. "Health education+"<br>29. "Education+"<br>30. "Intervention"
32. 29 OR 30 OR 31	28. 25 OR 26 OR 27	31. 28 OR 29 OR 30
33. 28 AND 32	29. 24 AND 28	32. 27 AND 31

34. 16 OR 33	30. 14 OR 29	33. 16 OR 32
35. Randomized controlled trial/<br>36. Randomized controlled trials as topic/<br>37. RCT<br>38. Random Allocation/<br>39. Controlled clinical trial/<br>40. Clinical trial/<br>41. Clinical trials as topic/<br>42. Trial*<br>43. Cohort studies/<br>44. Longitudinal studies/<br>45. Prospective studies/<br>46. Retrospective studies/<br>47. Follow-up studies/<br>48. Cross-sectional studies/<br>49. Clinical research	31. "Randomized controlled trial"<br>32. RCT<br>33. "Random allocation"<br>34. "Controlled clinical trial"<br>35. "Clinical trial"<br>36. Trial$<br>37. "Cohort stud?"<br>38. "Longitudinal stud?"<br>39. "Prospective stud?"<br>40. "Retrospective stud?"<br>41. "Follow-up stud?"<br>42. "Cross sectional stud?"<br>44. "Clinical research"	34. "Randomized controlled trials+"<br>35. "RCT"<br>36. "Clinical trials+"<br>37. "Community trials"<br>38. Trial*<br>39. "Cohort stud*"<br>40. "Longitudinal stud*"<br>41. "Prospective studies+"<br>42. "Retrospective stud*"<br>43. "Cross Sectional studies"<br>44. "Clinical research+"
50. 35 OR 36 OR 37 OR 38 OR 39 OR 40 OR 41 OR 42 OR 43 OR 44 OR 45 OR 46 OR 47 OR 48 OR 49 OR 50	45. 31 OR 32 OR 33 OR 34 OR 35 OR 36 OR 37 OR 38 OR 39 OR 40 OR 41 OR 42 OR 43 OR 44	45. 34 OR 35 OR 36 OR 37 OR 38 OR 39 OR 40 OR 41 OR 42 OR 43 OR 44
51. 11 AND 34 AND 50	46. 9 AND 30 AND 45	46. 11 AND 33 AND 45

2
